# MDA-5 Recognition of a Murine Norovirus

**DOI:** 10.1371/journal.ppat.1000108

**Published:** 2008-07-18

**Authors:** Stephen A. McCartney, Larissa B. Thackray, Leonid Gitlin, Susan Gilfillan, Herbert W. Virgin IV, Marco Colonna

**Affiliations:** Department of Pathology and Immunology, Washington University School of Medicine, St. Louis, Missouri, United States of America; University of North Carolina, United States of America

## Abstract

Noroviruses are important human pathogens responsible for most cases of viral epidemic gastroenteritis worldwide. Murine norovirus-1 (MNV-1) is one of several murine noroviruses isolated from research mouse facilities and has been used as a model of human norovirus infection. MNV-1 infection has been shown to require components of innate and adaptive immunity for clearance; however, the initial host protein that recognizes MNV-1 infection is unknown. Because noroviruses are RNA viruses, we investigated whether MDA5 and TLR3, cellular sensors that recognize dsRNA, are important for the host response to MNV-1. We demonstrate that MDA5−/− dendritic cells(DC) have a defect in cytokine response to MNV-1. In addition, MNV-1 replicates to higher levels in MDA5−/− DCs as well as in MDA5−/− mice *in vivo*. Interestingly, TLR3−/− DCs do not have a defect *in vitro*, but TLR3−/− mice have a slight increase in viral titers. This is the first demonstration of an innate immune sensor for norovirus and shows that MDA5 is required for the control of MNV-1 infection. Knowledge of the host response to MNV-1 may provide keys for prevention and treatment of the human disease.

## Author Summary

Gastroenteritis is a common disease in both developed and developing countries. The two main causes of this affliction are bacteria and viruses. The primary viruses implicated in gastroenteritis have been shown to be noroviruses, which include Norwalk virus, notorious for numerous recent outbreaks on cruise ships. We are interested in how the innate immune system detects viral infection and prepares the host to respond to the threat. To understand how the host responds to norovirus infection, we studied two classes of proteins, both of which are thought to detect signs of viral infection. We discovered that one of these proteins, melanoma differentiation associated protein-5 (MDA-5), is responsible for detecting a mouse norovirus that is genetically related to the human pathogen. These findings allow us to better understand the pathogenesis of norovirus infection and may provide clues for controlling the human disease.

## Introduction

Norwalk virus and other human noroviruses are common human pathogens responsible for most of the nonbacterial epidemic gastroenteritis in both developed and developing countries [1],[2],[3],[4],[5]. In humans, norovirus infection can result in vomiting, diarrhea, fever, malaise, and abdominal pain within 24 hours after infection. These symptoms usually clear within 48 hours, but the virus can persist asymptomatically for 3–6 weeks post-infection [6],[7]. Until recently the inability to culture human noroviruses has prevented investigation into its pathogenicity. The discovery and subsequent routine culture of murine norovirus-1 (MNV-1) has led to advances in understanding of both the norovirus lifecycle as well as the host response to norovirus infection [8],[9].

Noroviruses are in the *Calicivirus* family and are nonenveloped viruses containing a single-stranded positive-sense RNA genome. Norovirus genomes are covalently linked at the 5′ end to a viral nonstructural protein VPg [10]. Norovirus genomes encode three open reading frames (ORFs) [11],[12],[13],[14]. ORF1 encodes a polyprotein that is cleaved into at least six nonstructural proteins by the viral 3C-like protease [15],[16],[17],[18]. ORF2 encodes the major capsid protein, viral protein 1 [11],[19], while ORF3 encodes the small basic protein, viral protein 2 [20],[21]. An additional ORF, ORF4 was recently discovered in the MNV genome although the function of this ORF has yet to be characterized [14].

The rapid clearance of MNV-1 infection in immunocompetent mice indicates an important role for the innate immune system, since clearance precedes the timeframe normally associated with the initiation of adaptive immunity [22]. Previous work has revealed that MNV-1 infection of mice lacking either the type I and type II interferon (IFNα/β/γ) receptors or the STAT-1 molecule results in lethality [9],[22]. Several proteins are known to initiate the IFN response to viruses [23], including Toll-like receptors (TLR) [24], Rig-I-like helicases (RLH) [25],[26], PKR [27], and RNase L [28]. However, the initial sensor responsible for recognition of noroviruses and subsequent activation of cytokine response has not been determined.

TLRs are located on the plasma membrane and in endosomal compartments. Among the TLRs, TLR 7 and 8 recognize ssRNA [29],[30],[31], TLR9 recognizes DNA [32],[33], while TLR3 signals in response to dsRNA [34]. The RLHs are sensors located within the cytoplasm [26], which include Rig-I and MDA-5 [23],[35],[36] and signal through IPS-1/MAVS/Cardiff/VISA [37],[38],[39],[40]. Rig-I has recently been shown to preferentially recognize 5′-phosphorylated RNA [41],[42], while MDA5 responds to dsRNA [43]. Recently it has been shown that the lack of Rig-I does not confer susceptibility to human norovirus *in vitro* [44]. Because MDA5 [45],[46],[47],[48], and TLR3 [49],[50] have been shown to play a role in host response to other RNA viruses we investigated if these sensors might be involved in norovirus recognition *in vitro* and *in vivo* using the MNV-1 model system. In this study we demonstrate that indeed MDA5 is the predominant sensor of MNV-1 and initiates the innate immune response against the virus, and that TLR3 may also play a role in the response to MNV-1 in certain tissues.

## Results

### MDA-5 is required for cytokine response to MNV-1 by Bone Marrow-Derived DC

Previous studies have shown a requirement for the type I IFN response for control of MNV-1 infection *in vitro* [8]. Since both MDA5 and TLR3 have been shown to be involved in type I IFN and cytokine signaling in response to infection with other RNA viruses, we were interested to see if they may play a role in MNV-1 infection.

MNV-1 infection has a limited cell tropism- infecting only DC and macrophage lineages *in vitro* [8],[51]. In order to test whether the MDA5 or TLR3 sensors were important, BMDCs from Wild Type as well as TLR3−/− and MDA5−/− mice were cultured for 7 days and then inoculated with various MOI of MNV-1. After 24 hours supernatants from the *in vitro* infections were harvested and tested for cytokine secretion from the BMDCs.

**Figure 1:**
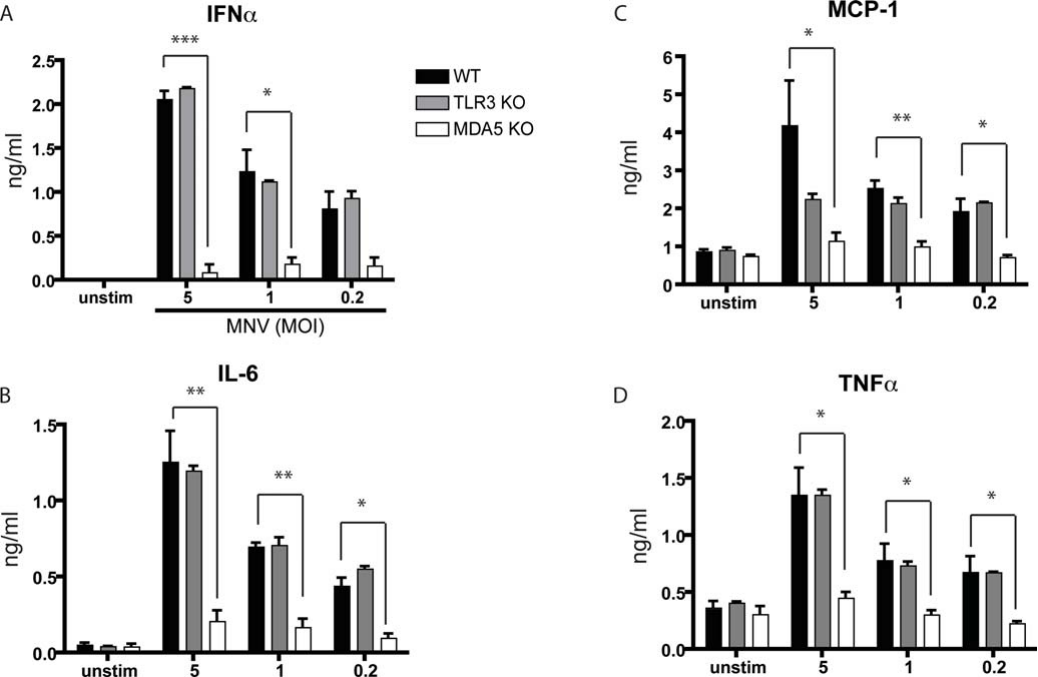
MDA5 is required for cytokine response to MNV *in vitro.* Bone marrow-derived dendritic cells from wild type (WT), TLR3 deficient (TLR3 KO), or MDA5 deficient (MDA5 KO) mice were infected *in vitro* with MNV at the indicated MOI. Cell culture supernatants were harvested 24 hours after inoculation, and examined for IFNα by ELISA (A) or for IL-6 (B), MCP-1 (C), or TNFa (D) by cytokine bead array. Data shown is the average of three independent experiments. Statistical analysis was done using student's t test where * = p<0.05, ** = p<0.01, and *** = p<0.001.

Interestingly, although WT and TLR3 DCs produced similar levels of IFNα and inflammatory cytokines in response to MNV stimulation, MDA5 deficient DCs produced significantly less IFNα, IL-6, MCP-1, TNFα (Figure 1) and IFNβ (data not shown). In this cell type MDA5 appears to be the primary sensor responsible for type 1 IFN production in response to MNV-1, however, we cannot rule out that other sensors may play a role in other cell types.

### MDA5 limits MNV-1 replication *in vivo*

MNV-1 infection naturally occurs after fecal-oral transmission [8]. In order to test whether MDA5 and TLR3 play a role in MNV-1 detection *in vivo* we infected WT, MDA5−/−, or TLR3−/− mice with MNV-1.CW3 perorally. Organs were then harvested from infected as well as mock-infected mice on days 1, 3, and 5 after inoculation and viral titers were determined for each sample.

**Figure 2:**
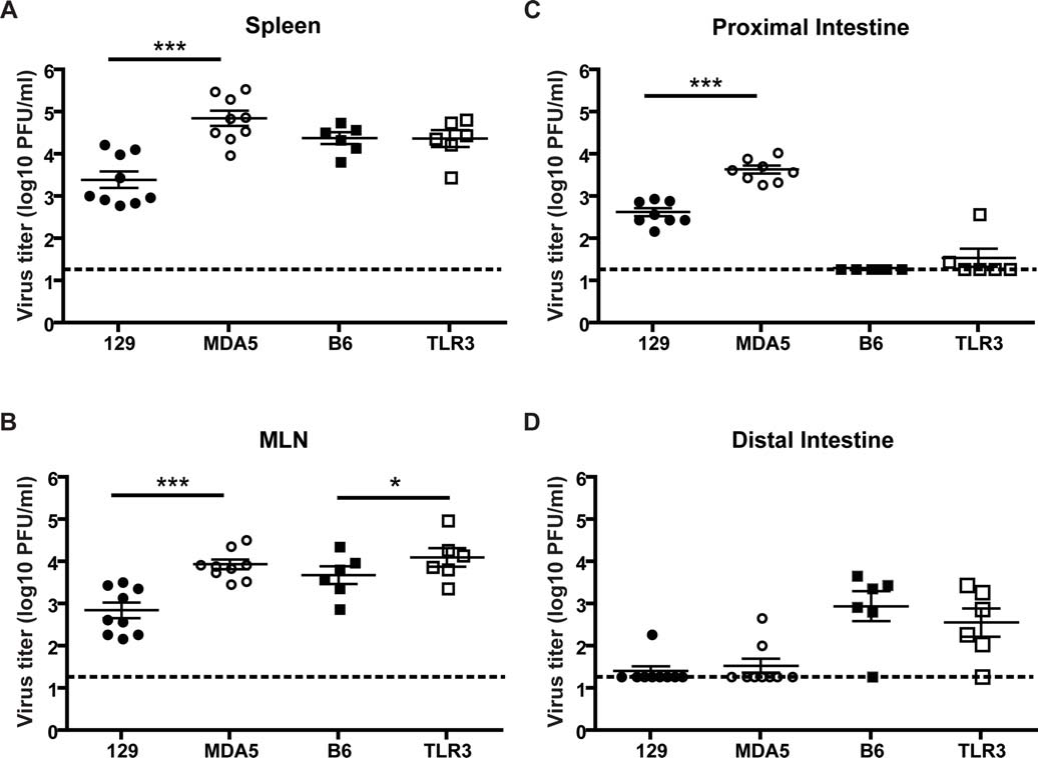
MNV replicates more efficiently in MDA5 and TLR3 KOs *in vivo*. Wild type (129 or B6), MDA5−/− (MDA5), or TLR3−/− (TLR3) mice were inoculated perorally with 3×10^7^ PFU MNV-1.CW3 or mock infected with media only. Organs were harvested 3 days after infection and viral titers of Spleen (A), Mesenteric Lymph Node (MLN) (B), Proximal Intestine (C), and Distal Intestine (D) were determined by plaque assay. Statistical significance was calculated using the Mann Whitney test, where *** = p<0.001, * = p<0.05. Mock-infected animals showed no detectable MNV-1 at all time-points tested. Data shown is from 6–9 animals. Dashed line indicates limit of detection of the assay.

MNV-1 titers were highest at d3 post infection. At this time-point MDA5−/− animals had significantly increased viral titers compared to wild type animals in the mesenteric lymph nodes, spleen, and proximal intestine (Figure 2). Minimal or no virus titers was detected by plaque assay in the distal intestine or feces (data not shown) in WT and MDA5−/− animals on the 129/Svj background. Interestingly, although there did not appear to be an effect of TLR3 deficiency in the detection of MNV-1 *in vitro*, there was a slight, but significant increase in viral titers in the mesenteric lymph node of TLR3−/− mice compared to wild type mice. No difference in virus titers was detected in the spleen or distal intestine between WT and TLR3−/− animals (Figure 2). This may indicate a role for TLR3 in MNV-1 detection in a tissue-specific manner. Minimal or negative titers were seen in proximal intestines and feces (data not shown) in WT and TLR3−/− animals on the B6 background. That B6 and 129 strains of mice have higher titers of virus in the distal and proximal intestine respectively has been reported in previous studies [14],[22] and may reflect the differential distribution of viral sensors along the gastrointestinal tract.

Previous work has demonstrated that lack of innate immune components leads to disseminated MNV-1 infection and is ultimately fatal [9]. Therefore we determined whether the lack of MDA5 could also cause a more extensive spread of MNV-1. However, we detected no virus titers in the lung or the liver in WT or MDA5−/− mice (data not shown). Consistent with the lack of systemic infection, serum samples taken from WT and MDA5−/− mice at 1, 3, and 5 days after inoculation were tested by ELISA for IFNα, IFNβ, and IFNγ and found to be negative (data not shown). The kinetics of clearance of MNV-1 infection was similar in WT and MDA5−/− mice *in vivo*, even though there was a significant increase in virus titers in the MDA5−/− animals at day 3. By day 5 post-inoculation no virus titers were detected in MDA5−/− spleen, MLN, or proximal intestine. In addition, there was no difference in survival between WT, MDA5−/−, or TLR3−/− mice for 35 days post-inoculation. In contrast, STAT-1−/− mice and IFNαβγR−/− mice have shown a survival defect to MNV-1 infection. Since these mice have complete abrogation of IFN signaling pathways, this may indicate that MDA5 and TLR3 are redundant or that other sensors may also play a role in MNV-1 detection.

### MNV-1 replicates more efficiently in MDA-5 deficient DCs

Because the MDA5−/− and TLR3−/− mice had an increase in MNV-1 virus titers, we wanted to test the cellular basis of this deficiency. To address this issue, we infected BMDCs from WT, MDA5−/−, and TLR3−/− mice with MNV-1 and harvested samples at 6-hour time-points after inoculation. The infections were done at a high and low MOI to test for effects on viral replication and spreading.

**Figure 3:**
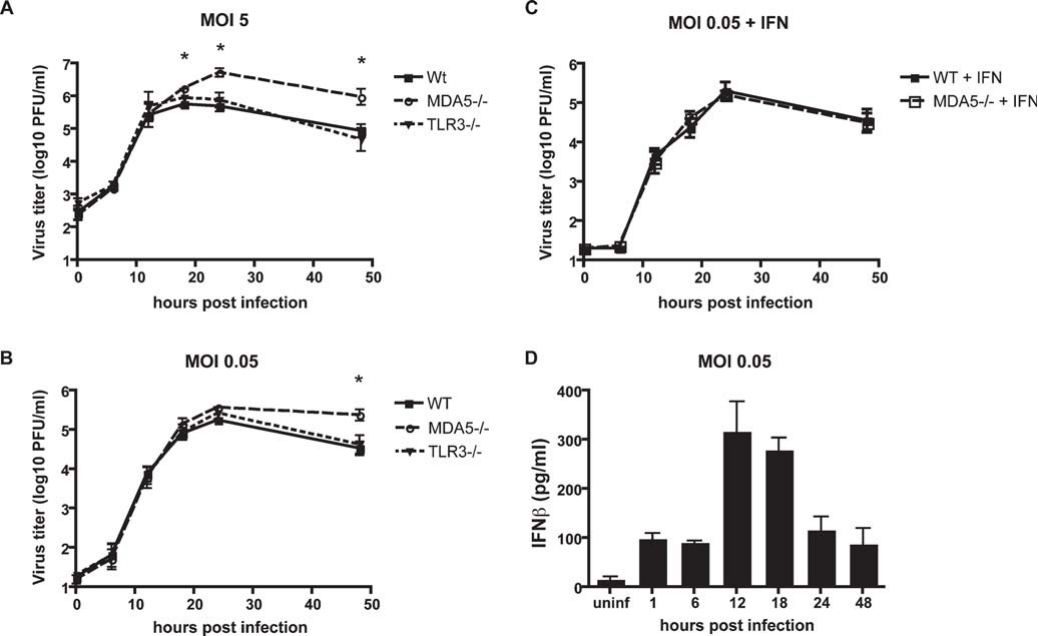
MDA5 deficiency leads to increased MNV titers *in vitro*. Bone marrow-derived dendritic cells from wild type (WT), MDA5−/−, or TLR3−/− mice were inoculated with MNV at an MOI of 5 (A) or 0.05 (B) or pre-treated with 20 U IFNα and then inoculated with an MOI of 0.05 (C). Viral titers were done at 6 hour time-points for each sample and statistical significance was determined using student's t test. There was no significant difference between WT and TLR3−/− titers, statistical significance is marked between WT and MDA5−/− titers where * = p<0.05. Data shown is the average of four independent experiments (A and B) or three independent experiments (C). In (D) supernatants from WT BMDC infected at MOI 0.05 were harvested at various time-points and tested for IFNβ by ELISA. Data shown is the average of three independent experiments.

Viral titers were identical in WT, MDA5−/−, and TLR3−/− mice up to 12 hours after inoculation at the high MOI (Figure 3a). However, starting at 18 hours pi, titers from MDA5−/− BMDCs began to increase over WT and TLR3−/− BMDCs, and leveled out to a significant difference at 24 and 48 hours, indicating that MDA5 recognition occurs late in viral infection. At a low MOI there was no significant difference between viral titers in WT and MDA5−/− cells until the 48 hour time-point (Figure 3b), indicating that the requirement for MDA5 recognition requires multiple cycles of replication. Indeed, the peak level of IFN production in WT BMDCs occurs at 12 hours post-infection after a low MOI inoculation (Figure 3d), suggesting that a cycle of viral replication is necessary to induce IFNβ. The increase in virus titers in the MDA5−/− cells reflects the defect in type I IFN produced in response to viral infection. IFN pretreatment of MDA5−/− BMDCs before infection with a low MOI reconstitutes the WT phenotype, preventing an increase in virus titers.

In both MOIs the kinetics of MNV-1 infection appears similar in WT and MDA5−/− BMDCs; the difference mainly appears to be in the total amount of viral replication seen in the MDA5−/− BMDCs. Interestingly, although TLR3−/− mice had an increase in MNV-1 titers, TLR3−/− BMDCs had no significant increase in titers. This may reflect a cell type-specific role for viral sensors.

### MDA-5 recognizes replication competent viral RNA

**Figure 4:**
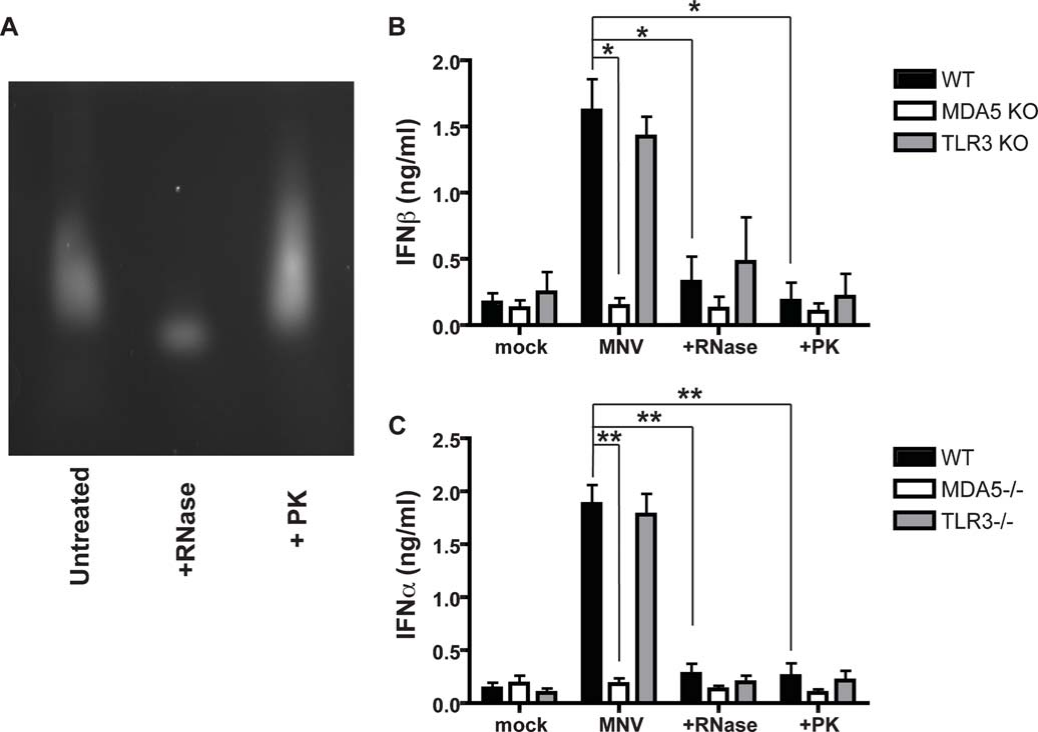
Proteinase K and RNase abrogates MDA5 recognition of viral RNA. WT, MDA5−/−, or TLR3−/− BMDCs were mock transfected or transfected with RNA purified from MNV virions that was treated with RNase A (RNase), Proteinase K (PK) or untreated. After 20 hours, supernatants were harvested and cytokines analyzed by ELISA for IFNβ (B) or IFNα (C). Data shown is from three independent experiments and statistics were calculated by student's t test, where * = p<0.05, ** = p<0.01. Treated and untreated RNA were electrophoresed on an agarose gel to visualize degradation (A).

Although we have demonstrated that MDA5 is required to recognize MNV-1, it is unclear which RNA feature is essential for recognition. Another sensor, Rig-I, recognizes viruses mainly through 5′-phosphorylation, however, in noroviruses this feature is absent because of a 5′ VPg cap [10]. To test whether 5′ RNA configuration is essential for MDA5 recognition we purified viral RNA from MNV-1 virions. BMDCs from WT, MDA5, or TLR3 deficient mice were then stimulated with the harvested RNA, as well as RNA treated with RNase A which degrades ssRNA, and proteinase K (PK), which degrades proteins- in this case the VPg cap. As expected, RNase treatment degraded the viral RNA, while PK treatment did not degrade the RNA as seen in Figure 4a.

Consistent with the results of *in vitro* MNV-1 infections shown in Figure 1, both WT and TLR3−/− BMDCs produced type 1 IFN in response to purified viral RNA, while MDA5−/− BMDCs had a significant decrease in IFN response (Figure 4b,c). However, the addition of PK or RNase to the RNA abrogated the cytokine response in WT and TLR3−/− BMDCs. This data demonstrates that VPg is required for MDA5 recognition of MNV-1, and suggests that MDA5 either directly recognizes RNA linked to VPg or, since VPg is required for norovirus replication [44], that MDA5 recognizes dsRNA generated during viral replication. Because MDA5 has been previously shown to recognize uncapped poly I∶C [46],[47], it is most likely that the result of PK treatment reflects the requirement for viral replication and the subsequent generation of dsRNA that is recognized by MDA5. Consistent with this hypothesis, WT BMDCs inoculated with UV-inactivated MNV-1 did not produce IFNβ (data not shown).

## Discussion

We have provided the first description of an initial sensor of norovirus infection. MDA5 recognizes MNV-1 and stimulates antigen presenting cells to produce type I interferon as well as IL-6, MCP-1, and TNFα that function to recruit other immune cells as well as activate antiviral pathways in host cells. Deficiency of this sensor results in lack of cytokine production as well as increased MNV-1 replication in deficient cells and mice.

It is interesting to note that although MDA5 deficient cells have a severe defect in IFNα production, MDA5−/− mice contain and clear MNV-1 infection. This is in contrast the severe systemic infection and survival phenotype as the IFNαβγR or STAT1 deficient mice, which lack type I and type II IFN signaling pathways. STAT-1−/− and IFNαβγR−/− mice have a 4 log increase in viral titers *in vivo* and a 2 log increase in viral titers *in vitro* as seen in previously published data [22]. In our study MDA5−/− mice have a 1-log increase in viral titers *in vivo* and *in vitro*, while TLR3−/− mice have a 0.5 log increase, but only in one organ *in vivo*. This indicates to us that although MDA5 may be the dominant sensor in BMDCs, it is likely that in other cell types additional sensors can detect MNV-1, such as Rig-I, PKR, TLR7, and perhaps other unknown sensors. Further investigation is needed to determine if mice and cells that are deficient in multiple nucleic acid sensors lack all ability to respond to MNV-1 and whether they therefore have a more severe phenotype. Data from our lab and others [44] from *in vitro* experiments suggest that lack of TLR3 and Rig-I seem to have little effect on MNV-1 recognition individually, however, we cannot rule out that their involvement is masked by MDA5.

Although the putative recognition structure for Rig-I has previously been determined [41],[42], the RNA structure recognized by MDA5 in viral infection remains unclear. We demonstrated that MDA5 recognition of MNV RNA is abrogated by treatment with PK, which degrades VPg, preventing viral replication. This data suggests that VPg is essential for MDA5 recognition of MNV-1. Although we cannot rule out the possibility that MDA5 recognizes the VPg-RNA structure itself, this is less likely because MDA5 is known to respond to poly I∶C which has no protein cap. It is more likely that since VPg is essential for viral replication of the ssRNA norovirus genome, loss of VPg prevents MDA5 recognition of dsRNA produced during viral replication. Learning more about which viruses are recognized by MDA5 may provide hints as to what this protein recognizes. This information could then be used to design adjuvants to manipulate the immune response for both vaccine design as well as in treatment of viral infection.

## Materials and Methods

### Cell lines

RAW264.7 cells were maintained in Dulbecco modified Eagle medium (DMEM) supplemented with 10% fetal calf serum (Hyclone), 100 U penicillin/ml, 100 µg/ml streptomycin, 10 mM HEPES, and 2 mM L-glutamine.

### Viruses

All experiments were performed with MNV-1.CW3 [14]. Virus stocks were generated using RAW 264.7 cells that were inoculated with a multiplicity of infection (MOI) of 0.05 in VP-SFM media (Gibco) and harvested approximately 40 hours after inoculation. Infected cell lysates were frozen at −80°C and thawed three times. Cell lysates were clarified by low-speed centrifugation for 20 min at 3,000 rpm. To generate a concentrated virus stock, clarified cell lysates were concentrated by centrifugation at 4°C for 3 h at 27,000 rpm (90,000 g) in a SW32 rotor.

### Bone marrow-derived DC

Bone marrow was flushed from the femurs of mice and cultured as described previously [52]. Briefly, cells were cultured in RPMI (Gibco) with 10% fetal calf serum (Hyclone), Glutamax, Na Pyruvate, Non-Essential AAs, and Kanamycin for 7–8 days at 37 degrees.

### Mice

MDA5−/− mice were described previously [47]. For the infection studies mice backcrossed onto a pure 129/SVJ background were used. Control WT mice were age and sex matched and were obtained from littermate controls and from Jackson Lab for 129/SVJ and C57BL/6. TLR3−/− mice were kindly provided by Richard Flavell [34]. All mice were bred and housed in a pathogen free facility and regularly tested for MNV-1 antibodies.

### *In vitro* stimulations

BMDCs were counted and plated at 200,000 cells/well in a 96 well plate. MNV-1 was added at various MOI to the cultures, or alternatively 500 ng RNA was complexed with lipofectamine 2000 (invitrogen) and added according to manufactures instructions. After 20–24 hours supernatants were harvested and stored at −20 degrees until cytokine analysis. IFNα and IFNβ levels from the supernatants were measured by ELISA (PBL Biomedical Laboratory, New Brunswick, NJ), while IL-6, MCP-1, and TNFα levels were determined by cytokine bead array (BD Biosciences).

### *In vivo* infections

WT, TLR3−/−, or MDA5−/− mice were infected perorally with 3×10^7^ PFU MNV1.CW3 [14] or mock-infected with media only. Three days post-infection the following organs were harvested and stored at −80 degrees until assayed: spleen, liver, mesenteric lymph node, lung, proximal intestine, distal intestine, stool, and serum.

### MNV-1 plaque assay

Tissue samples were homogenized in 1 ml complete DMEM by bead beating with 1.0-mm zirconia/silica beads (BioSpec Products, Inc.). Tissue homogenates were diluted 1∶10 in complete DMEM and tested for viral titers by using a plaque assay that has been previously described [8]. Briefly, 2×10^6^ RAW264.7 cells were seeded into each well of six-well plates, and infected the next day with 10-fold dilutions of tissue homogenate in duplicate. After a 1-hr infection, the inoculum was removed and wells were overlaid with 1.5% SeaPlaque agarose (Cambridge Biosciences) in complete minimal essential medium and incubated at 37°C. After 48 hrs, a second overlay was added containing 1.5% SeaKem agarose (Cambridge Biosciences) and 0.01% neutral red in complete minimal essential medium. After 8 hrs, plaques were then visualized.

### RNA preparation

Total viral RNA was harvested from concentrated virus stock using Trizol reagent (Invitrogen) according to manufacturer's instructions. Purified RNA was incubated with either 10 units RNase A (Sigma) in NEB buffer 3 (New England Biolabs) or with 200 µg/ml proteinase K (Sigma) in 0.1 M NaCl, 10 mM Tris (pH 8), 1 mM EDTA, 0.5% sodium dodecyl sulfate or left untreated in NEB buffer 3 for 30 minutes at 37°C then stopped with 0.1 mM EDTA. To test for RNA degradation, samples were run on a 1% agarose gel and visualized using a UV light box.
