# Correction: MDA-5 Recognition of a Murine Norovirus

**DOI:** 10.1371/annotation/3ce83911-9ccf-4452-a690-2816d0e94c10

**Published:** 2008-10-10

**Authors:** Stephen A. McCartney, Larissa B. Thackray, Leonid Gitlin, Susan Gilfillan, Herbert W. Virgin, Marco Colonna

The fifth author's name is displayed incorrectly in the by-line and in the article citation. The author's correct attribution is Herbert W. Virgin. The correct citation is: McCartney SA, Thackray LB, Gitlin L, Gilfillan S, Virgin HW, et al. (2008) MDA-5 Recognition of a Murine Norovirus. PLoS Pathog 4(7): e1000108. doi:10.1371/journal.ppat.1000108

